# Teratogenic Potential of Antiepileptic Drugs in the Zebrafish Model

**DOI:** 10.1155/2013/726478

**Published:** 2013-11-14

**Authors:** Sung Hak Lee, Jung Won Kang, Tao Lin, Jae Eun Lee, Dong Il Jin

**Affiliations:** Department of Animal Science & Biotechnology, Chungnam National University, Daejeon 305-764, Republic of Korea

## Abstract

The zebrafish model is an attractive candidate for screening of developmental toxicity during early drug development. Antiepileptic drugs (AEDs) arouse concern for the risk of teratogenicity, but the data are limited. In this study, we evaluated the teratogenic potential of seven AEDs (carbamazepine (CBZ), ethosuximide (ETX), valproic acid (VPN), lamotrigine (LMT), lacosamide (LCM), levetiracetam (LVT), and topiramate (TPM)) in the zebrafish model. Zebrafish embryos were exposed to AEDs from initiation of gastrula (5.25 hours post-fertilization (hpf)) to termination of hatching (72 hpf) which mimic the mammalian teratogenic experimental design. The lethality and teratogenic index (TI) of AEDs were determined and the TI values of each drug were compared with the US FDA human pregnancy categories. Zebrafish model was useful screening model for teratogenic potential of antiepilepsy drugs and was in concordance with *in vivo* mammalian data and human clinical data.

## 1. Introduction

Antiepileptic drug (AED) exposure *in utero* has been associated with major congenital malformations (MCMs) and adverse cognitive outcomes in the offspring of women with epilepsy (WWE) [1, 2]. The use of older-generation AEDs during pregnancy is known to be associated with a two- to threefold increased risk of birth defects in the offspring and possibly also other adverse outcomes in the exposed infant. Much less has been known about newer-generation AEDs [3].

Preclinical toxicological studies include testing for teratogenicity in at least two different species. However, such animal teratology studies are generally expensive and time consuming [4]. Clinical studies on the teratogenic effects of AEDs have been too small and underpowered to enable researchers to draw significant conclusions [5].

There is a need to develop a teratogenicity assay for its ability to predict the teratogenic potential of drugs. The criteria for a useful teratogenicity screening should include cost-effectiveness, adequate throughput, straightforward assay conduct, reproducibility, and concordance with *in vivo* mammalian data [4, 6]. 

Zebrafish are inexpensive and easy to maintain and breed in large numbers [7, 8]. Furthermore, zebrafish development is similar to that of mammals, and many molecular pathways are evolutionarily conserved between zebrafish and humans [4]. Owing to these advantages, zebrafish embryo is considered a suitable alternative model for traditional *in vivo* developmental toxicity screening [9]. 

In 1975, the FDA created guidelines for drug companies to follow in regard to labeling medications about their effects on reproduction and pregnancy. The pregnancy category of a pharmaceutical agent is an assessment of the risk of fetal injury due to the pharmaceutical. The FDA has a categorization of drug risks to the fetus that runs from “Category A” to “Category X” [10]. 

In this study, we evaluated the teratogenic potential of seven AEDs: carbamazepine (CBZ, FDA drug pregnancy category “D”), ethosuximide (ETX, “C”), valproic acid (VPN, “D”), lamotrigine (LMT, “C”), lacosamide (LCM, “C”), levetiracetam (LVT, “C”), and topiramate (TPM, “D”) in the zebrafish model. Zebrafish embryos were exposed to AEDs from initiation of gastrula (5.25 hours post-fertilization (hpf)) to termination of hatching (72 hpf) which mimic the exposure time of mammalian teratogenic experimental design. The lethality and teratogenicity were determined and used to calculate the teratogenic index (TI). To evaluate the concordance of the TI values in zebrafish model with *in vivo* mammalian data and human clinical data, we compared the correlation TI values in zebrafish embryos with FDA categories of seven AEDs. 

## 2. Materials and Methods

### 2.1. Animals

Adult zebrafish (wild-type AB strain) of either sex were obtained from a commercial supplier (OK aqua-mall, Gyeonggi-Do, Korea). Zebrafish were housed separately by gender under a 14 h light/10 h dark cycle and fed live brine shrimp 2-3 times a day. The water temperature was maintained at 28 ± 1°C and pH 7. The day before spawning, two pairs of adult zebrafish were placed in a breeding tank equipped with a spawning tray. Eggs were collected and placed in Petri dishes filled with egg water (60 *μ*g ocean salt/mL) [8]. Shortly after spawning, eggs were collected from the cage, and fertilized eggs were selected for all experiments. All animal care and use procedures were approved by the Institutional Animal Care and Use Committee of Chungnam National University.

### 2.2. Test Drugs

Drugs were purchased from Sigma-Aldrich (St. Louis, MO, USA) and Hanchem Co., Ltd. (Daejeon, Korea). Carbamazepine (CBZ, CAS no. 298-46-4, purity 100%), ethosuximide (ETX, CAS No. 77-67-8, purity 99.9%), and valproic acid sodium salt (VPN, CAS no. 1069-65-5, purity 99.9%) were purchased from Sigma-Aldrich, and lamotrigine (LMT, CAS no. 84057-84-1, purity > 97%), lacosamide (LCM, CAS no. 175481-36-4, purity > 97%), levetiracetam (LVT, CAS no. 102767-28-2, purity > 97%), and topiramate (TPM, CAS no. 97240-79-4, purity > 97%) were purchased from Hanchem Co., Ltd.

### 2.3. Drug Exposure of Zebrafish Embryos

CBZ, LCM, LMT, and TPM were dissolved in DMSO (Sigma-Aldrich, St. Louis, MO, USA) and the remaining drugs dissolved in egg water. Typically, 5 to 6 selected embryos were transferred to 24 multiwell plates (Becton Dickinson, Franklin Lakes, NJ, USA). DMSO (10 *μ*L) or egg water solution (50 *μ*L) was added to 1 mL aliquots of egg water. DMSO (1%, v/v) served as the control solution. 

**Figure 1:**
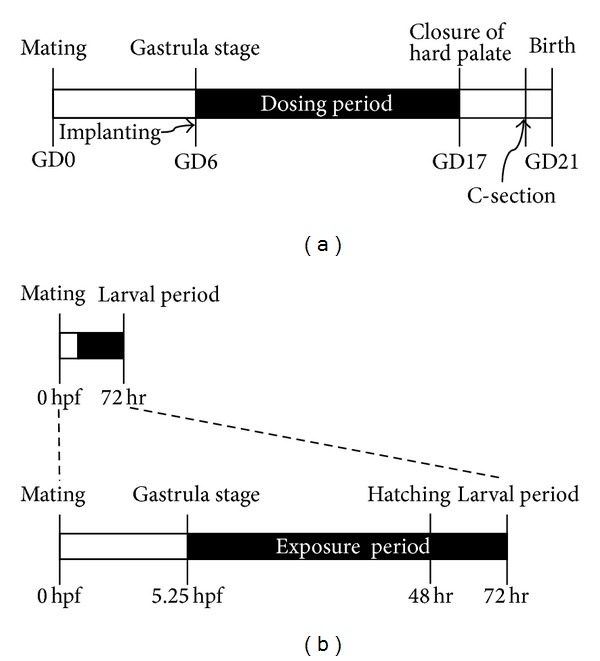
The exposure time of test compounds was modified to concur with those of the rodent teratogenicity study. (a) Typical experimental method for rat. GD: gestation day. (b) Modified method for zebrafish. hpf: hours post-fertilization.

Embryos were exposed to test compounds from initiation of gastrula (5.25 hpf) to termination of hatching (72 hpf) [11]. This exposure duration mimics that of rodent embryonic development (implantation to closure of the hard palate) (Figure 1).

### 2.4. Evaluation of Lethality and Teratogenic Effects

Endpoints were combined and modified based on the procedures of [4, 12, 13]. Embryos were examined daily and evaluated at 72 hpf. Lethal or teratogenic effects were recorded under an Olympus SZ61 stereomicroscope (Tokyo, Japan). The 8 hpf time point served as a control step to identify unfertilized eggs. 

Lethality endpoints (coagulation of the embryo, nondetection of the heartbeat) and teratogenicity endpoints (malformation of the head, tail, or heart, scoliosis, deformity of yolk, and growth retardation) were evaluated under a microscope at 72 hpf.

### 2.5. Calculation of the Teratogenicity Index (TI)

In order to characterize the teratogenic potential of a test substance, the teratogenicity index (TI), which was defined as the quotient of LC_50_ and EC_50_, was calculated [12, 14].

### 2.6. Statistical Analysis

Egg batches were only used at fertilization rates of ≥90%. An assay was considered valid if the controls did not show >10% teratogenic plus lethal effects at 72 hpf. LC_50_ and EC_50_ values were measured with PHARM/PCS (Version 4, Murray Springer-Verlag). The teratogenicity was analyzed using one-way analysis of variance (ANOVA), followed by Dunnett's multiple comparison test (Version 5.0, GraphPad Prism for Windows).

## 3. Results

Zebrafish embryos were exposed to AEDs from initiation of gastrula (5.25 hpf) to termination of hatching (72 hpf). The concentrations of each AED were chosen based on preliminary experiments (data not shown) and their feasible solubility. All controls fulfilled the acceptance criteria, specifically, ≥90% fertilization rate and ≤10% teratogenic effect. 

### 3.1. Lethal and Teratogenic Effects of Antiepileptic Drugs

**Figure 2:**
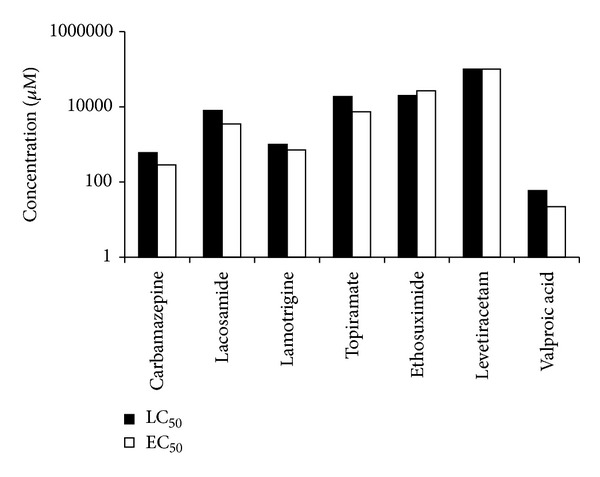
Lethal and teratogenic effects of antiepileptic drugs on zebrafish embryos at 72 hpf.

The lethality and teratogenicity of antiepileptic drugs were determined and calculated as TI at 72 hpf. Results are presented in Figure 2. The LC_50_ and/or EC_50_ values of LMT and LVT could not be calculated based on the solubility limit. TI values were ranked as follows: VPN > TPM > LCM > CBM > LMT > LVT ≅ ETX. 

### 3.2. Malformation Effects of Antiepileptic Drugs

**Figure 3:**
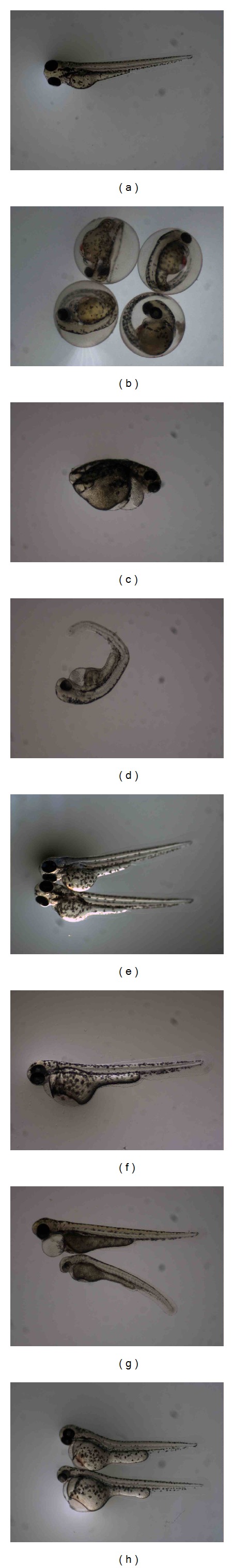
Zebrafish Embryos at 72 hpf. (a) Normal, 1% DMSO (×25), (b) 1 mM Carbamazepine (×30), (c) 5 M Valproic acid (×30), (d) 100 *μ*M Lamotrigine (×35), (e) 1 mM Levetiracetam (×25), (f) 500 *μ*M Lacosamide (×30), (g) 10 mM Ethosuximide (×30) and (h) 5 mM Topiramate (×25).

The teratogenicity endpoints (malformation of head, tail, or heart, scoliosis, deformity of yolk, and growth retardation) were evaluated under a microscope at 72 hpf (Figure 3). Data are presented in Table 1. 

LCM and LMT induced several kinds of malformations and showed significant difference between dose levels. There was some specific type's induction of malformation according to drugs. The main malformation of VPN was growth retardation, and TPM-induced multimalformation included heart edema, yolk deformity, and scoliosis.

### 3.3. Comparison of TI and Human Pregnancy Categories

The calculated TI values of the compounds were compared with the US FDA human pregnancy category (Table 2, Figure 2). Three drugs CBM, TPM, and VPN, which were classified as pregnancy category D by FDA and showed T1 values greater than 2, while the four drugs, ETX, LMT, LCM, and LVT, which were classified as category C by FDA, showed a wide range of T1 values between 0.76 and 2.3. 

## 4. Discussion

Standard developmental toxicity studies are generally expensive and time-consuming and combinations of antiepileptic drugs are not tested preclinically; the use of zebrafish has the potential to provide a level of predictivity that is as good as or better than that of these current models [15]. 

It was reported that zebrafish model was very successful in discriminating between teratogens and nonteratogens, having an 87% concordance with *in vivo* mammalian data and both a low false-positive and low false-negative rates of 15 and 11%, respectively [4]. The concordance between zebrafish embryo and mammalian models of developmental toxicity ranges from 64% to 100% [9]. The teratogenic potential of compounds can be predicted quantitatively by ranking zebrafish embryos based on a scoring system for phenotypic changes that is conceptually similar to morphological assessments conducted using *in vivo* embryo-fetal development of mammals [15]. 

The teratogenicity of antiepileptic drugs is a well-defined subject. The incidences of major malformations include spina bifida, cleft palate, limb reduction defects, cardiac abnormalities, hypospadias, and gastrointestinal atresia [16]. The exact mechanism by which the AEDs mediate abnormalities in the fetus is uncertain. VPN, which was known as inhibitior of histone deacetylases (HDACs), was well investigated and known as generate malformations such as edema, brain deformities, a shortened and bent tail, and bipartite axiation of the posterior trunk in zebrafish. The effects of zebrafish were similar to those observed in mammals [17].

The effects of teratogenic agents on developing organs are susceptible to the developmental stage at the time of exposure. There are critical periods of susceptibility to organ systems affected by these agents. In rat teratogenic study, test compounds are commonly exposed from implantation to closure of the hard palate during which major organ formation occurs, and in zebrafish, rapid morphogenesis is completed at hatching stage which is a similar stage to rat development [18]. So we observed zebrafish embryos at 72 hrs and collected malformations data in this study. 

In this experiment, we tried to discriminate the embryotoxic effect (lethality) and malformation effect (teratogenicity) of 7 AEDs in zebrafish model. The 7 AEDs showed large range of LC_50_ and EC_50_. In VPN, the LC_50_ was 59 *μ*M, which was the lowest lethal concentration, and the E_50_ was 22 *μ*M, whose observed teratogenic effect was mainly growth retardation. The other side, the LC_50_ of LVT, was over 100 mM, and the EC_50_ of LVT was over 100 mM, which did not increase teratogenic effect over lethal dose. 

Based on LC_50_ and EC_50_ values, a teratogenic index (TI) was calculated. A greater TI value is associated with a toxic agent that produces wide separations between the malformation and lethality dose-response curves. It is possible to have a toxic agent that causes severe malformations but not mortality; conversely, a potentially developmental toxic chemical can be so lethal that malformations are not observed [14]. Three drugs which were classified as pregnancy category D by FDA showed greater than 2 of TI values. But the other four drugs, which were classified as category C by FDA, showed a wide range of TI values between 0.76 and 2.3. 

Although zebrafish have been extensively used as a model in toxicity testing, they were relatively uninformative for understanding the underlying biological complexity and for reducing the uncertainties in predicted outcomes, especially in humans, due to (1) species differences, (2) variability in outcomes, and (3) uncertainties in extrapolating outcomes from the high-dose, short-term animal bioassay exposure regimens to the more common low-dose chronic exposure scenarios experienced in humans. 

In our experiments, three compounds classified as pregnancy category D showed TI values greater than 2. The sensitivity of this study model to humans is 100% (3/3). Zebrafish model could support teratogenic screening model as an alternative model for developmental toxicity studies to predict effects in humans. Zebrafish model was useful screening model for teratogenic potential of antiepilepsy drugs and was in concordance with *in vivo* mammalian data and human clinical data.

## Figures and Tables

**Table 1:** Malformation effects of antiepileptic drugs on zebrafish.

	Embryo numbers (*n*)			Malformation in embryos (*n*)							
	Treated	Lethal (%)	Normal (%)	Observed	Head	Tail	Heart	Yolk	Scoliosis	Growth retardation^1^	Teratogenicity
Carbamazepine											
2 mM	10	10 (100%)	—	0	—	—	—	—	—	—	—
1 mM	16	12 (75%)	0 (0%)	4	0 (0%)	0 (0%)	0 (0%)	0 (0%)	0 (0%)	4 (100%)	4 (100%)
0.5 mM	19	10 (53%)	1 (11%)	9	0 (0%)	0 (0%)	4 (44%)	0 (0%)	1 (11%)	6 (67%)	8 (89%)
0.25 mM	10	2 (20%)	5 (50%)	8	0 (0%)	0 (0%)	2 (25%)	0 (0%)	0 (0%)	1 (13%)	3 (38%)
0.1 mM	20	7 (35%)	9 (45%)	13	0 (0%)	1 (8%)	3 (23%)	0 (0%)	1 (8%)	2 (15%)	4 (31%)

Lacosamide											
10 mM	14	7 (50%)	0 (0%)	7	0 (0%)	0 (0%)	0 (0%)	0 (0%)	0 (0%)	7 (100%)	7 (100%)^b^
5 mM	26	14 (54%)	2 (8%)	12	0 (0%)	2 (17%)	7 (58%)	0 (0%)	4 (33%)	3 (25%)	10 (83%)^b^
2.5 mM	10	3 (30%)	3 (30%)	7	0 (0%)	0 (0%)	1 (14%)	0 (0%)	0 (0%)	3 (43%)	4 (57%)^b^
1 mM	26	5 (19%)	16 (62%)	21	0 (0%)	1 (5%)	1 (5%)	0 (0%)	1 (5%)	3 (14%)	5 (24%)
0.5 mM	25	4 (16%)	20 (80%)	21	0 (0%)	1 (5%)	1 (5%)	0 (0%)	1 (5%)	1 (5%)	2 (10%)^a^

Lamotrigine											
1 mM	25	5 (20%)	7 (28%)	20	1 (5%)	1 (5%)	8 (40%)	6 (30%)	7 (35%)	5 (25%)	13 (65%)^b^
0.5 mM	25	6 (24%)	10 (40%)	19	1 (5%)	2 (11%)	4 (21%)	5 (26%)	2 (11%)	2 (11%)	9 (47%)
0.25 mM	15	2 (13%)	12 (80%)	13	0 (0%)	1 (8%)	0 (0%)	1 (8%)	0 (0%)	0 (0%)	1 (8%)
0.1 mM	25	9 (36%)	12 (48%)	16	0 (0%)	0 (0%)	3 (19%)	2 (13%)	0 (0%)	0 (0%)	4 (25%)
0.05 mM	25	9 (36%)	16 (64%)	16	0 (0%)	0 (0%)	0 (0%)	0 (0%)	0 (0%)	0 (0%)	0 (0%)^a^

Topiramate											
50 mM	10	8 (80%)	0 (0%)	2	0 (0%)	0 (0%)	2 (100%)	2 (100%)	1 (50%)	1 (50%)	2 (100%)
10 mM	16	10 (63%)	4 (25%)	6	0 (0%)	0 (0%)	2 (33%)	1 (17%)	2 (33%)	1 (17%)	2 (33%)
5 mM	15	3 (20%)	5 (33%)	12	0 (0%)	0 (0%)	4 (33%)	1 (8%)	1 (8%)	3 (25%)	7 (58%)
1 mM	14	5 (36%)	3 (21%)	9	0 (0%)	0 (0%)	6 (67%)	2 (22%)	2 (22%)	2 (22%)	6 (67%)
0.5 mM	15	3 (20%)	9 (60%)	12	0 (0%)	1 (8%)	3 (25%)	0 (0%)	0 (0%)	0 (0%)	3 (25%)

Ethosuximide											
100 mM	15	15 (100%)	—	0	—	—	—	—	—	—	—
50 mM	15	9 (60%)	2 (0%)	6	0 (0%)	0 (0%)	0 (0%)	0 (0%)	0 (0%)	4 (67%)	4 (67%)
10 mM	15	7 (47%)	1 (7%)	8	0 (0%)	0 (0%)	7 (88%)	0 (0%)	0 (0%)	6 (75%)	7 (88%)
5 mM	15	6 (40%)	9 (60%)	9	0 (0%)	0 (0%)	0 (0%)	0 (0%)	0 (0%)	0 (0%)	0 (0%)
1 mM	15	6 (40%)	9 (60%)	9	0 (0%)	0 (0%)	0 (0%)	0 (0%)	0 (0%)	0 (0%)	0 (0%)

Levetiracetam											
100 mM	14	6 (43%)	7 (50%)	8	0 (0%)	1 (13%)	0 (0%)	0 (0%)	0 (0%)	1 (13%)	1 (13%)
50 mM	15	6 (40%)	9 (60%)	9	0 (0%)	0 (0%)	0 (0%)	0 (0%)	0 (0%)	0 (0%)	0 (0%)
10 mM	10	5 (50%)	5 (50%)	5	0 (0%)	0 (0%)	0 (0%)	0 (0%)	0 (0%)	0 (0%)	0 (0%)
5 mM	15	6 (40%)	9 (60%)	9	0 (0%)	0 (0%)	0 (0%)	0 (0%)	0 (0%)	0 (0%)	0 (0%)
1 mM	15	7 (47%)	7 (47%)	8	0 (0%)	0 (0%)	1 (13%)	0 (0%)	0 (0%)	0 (0%)	1 (13%)

Valproic acid											
100 *μ*M	12	12 (100%)	—	0	—	—	—	—	—	—	—
50 *μ*M	20	3 (15%)	4 (20%)	17	—	—	—	—	—	13 (76%)	13 (76%)^b^
25 *μ*M	20	2 (10%)	1 (5%)	18	—	—	—	—	—	17 (94%)	17 (94%)^b^
12.5 *μ*M	20	3 (15%)	13 (65%)	17	—	—	—	—	—	4 (24%)	4 (24%)^a^
6.25 *μ*M	20	3 (15%)	15 (75%)	17	—	—	—	—	1 (5%)	2 (12%)	2 (12%)^a^

1% DMSO	28	2 (7%)	26 (93%)	28	0 (0%)	0 (0%)	1 (4%)	0 (0%)	1 (4%)	0 (0%)	2 (7%)

1: including unhatched embryo at 72 hours post-fertilization.

“—” Indicated the numbers not determined.

^a,b^Different characters indicate significant difference within drugs (<0.05).

**Table 2:** Comparison of human pregnancy category and zebrafish TI values.

Compound	Human pregnancy category by FDA	Zebrafish TI
Carbamazepine	D	2.1
Lacosamide	C	2.3
Lamotrigine	C	>1.4
Topiramate	D	2.5
Ethosuximide	C	0.76
Levetiracetam	C	<1
Valproic acid	D	2.68
